# Improved bivariate analysis of canola survivability against blackleg disease

**DOI:** 10.1007/s00122-025-04993-x

**Published:** 2025-08-22

**Authors:** Timothy Thavarajah, James Walter, Julian Taylor

**Affiliations:** 1 School of Agriculture, Food and Wine, The University of Adelaide, Waite Campus, Glen Osmond, SA 5064 Australia; 2 Australian Grain Technologies Pty Ltd, 20 Leitch Road, Roseworthy, SA 5371 Australia

## Abstract

*****Key message***:**

**The bivariate analysis of canola survivability against blackleg disease with marker-based genomic information, a flexible residual variance model, and a novel selection measure can improve genetic gain for blackleg resistance.**

****Abstract**:**

Canola (*Brassica napus*) is an important oilseed crop grown extensively worldwide. It is deleteriously affected by the pathogen *Leptosphaeria maculans*, commonly known as blackleg, causing up to 15% yield loss in Australia annually. The most effective way to manage this disease is by growing resistant varieties. Screening genotypes for blackleg resistance has typically involved deriving percentage survivability against blackleg (from plant counts at emergence and maturity) and conducting a univariate analysis. More comprehensive approaches have involved a bivariate analysis that accounts for the correlation between plant counts. In this research, we have collated a new dataset from disease nurseries within a commercial breeding programme, comprised of related genotypes evaluated over 3 years at four locations across Australia, and outlined an innovative bivariate analysis approach. The research objectives were to (1) incorporate genomic marker information; (2) apply a more flexible residual model; and (3) develop a novel selection measure, responsiveness to blackleg disease, from the bivariate regression. Moderate to strong genetic correlations were found between traits, ranging between 0.49 and 0.91. The incorporation of genomic markers benefitted the maturity count more than emergence count. Furthermore, the more flexible residual model significantly improved model fit in five experiments. Using responsiveness as a selection measure produced comparable rankings with the univariate analysis of per cent survivability, with some re-ranking of genotypes which reflects the improved analysis through the bivariate approach. Ultimately, these results demonstrate an improvement over historic analyses, thus encouraging their adoption in canola breeding programmes to accelerate genetic gain for blackleg resistance.

**Supplementary Information:**

The online version contains supplementary material available at 10.1007/s00122-025-04993-x.

## Introduction

*Brassica napus L.* (rapeseed or canola) is the second most widely cultivated oilseed globally after soybean (Amas et al. 2021). With rising demand for high-quality plant-based oils, animal feed, and biofuels, the significance of canola towards the agricultural industry and economy is expected to grow (Fikere et al. 2018; Laviola et al. 2022). The intensification of canola cropping has however led to an increase in prevalence of plant diseases, adversely affecting canola production worldwide (Kirkegaard et al. 2021). Blackleg disease, caused by the hemibiotrophic fungal pathogen *Leptosphaeria maculans*, is the most destructive disease in canola (Amas et al. 2021). In Australia, it results in an estimated yield loss of 10 to 15% annually, with severe cases reaching up to 90% (Sprague et al. 2006; Van de Wouw et al. 2016). Blackleg’s high evolutionary potential enables it to quickly adapt to high selection pressure, leading to the rapid breakdown of disease resistance in commercial canola varieties, often known as the ‘boom and bust’ cycle (Marcroft et al. 2012; McDonald and Linde 2002; Van de Wouw et al. 2014). As such, effective management strategies are crucial in minimising the impact of blackleg on crop yield. Currently, the most common strategies include growing resistant varieties, fungicide application, and changes in farming management practices (Amas et al. 2021; Van de Wouw et al. 2021). Among these, the cultivation of genetically resistant varieties has been considered the most sustainable and effective management of blackleg disease for canola growers (Amas et al. 2021; Potter et al. 2016).

Through the advancement of genomic selection (GS) coupled with a decline in genotyping costs, plant breeders have been able to accurately select for superior crop varieties based on their genetic makeup with genome-wide markers (Meuwissen et al. 2001; Heffner et al. 2010). This significantly accelerates genetic gain by shortening breeding cycles and increasing selection accuracy for desirable traits (Fikere et al. 2018). Historically, breeding programmes have relied on univariate (single trait) GS due to its simplicity, computational efficiency, and ease of interpretation (Montesinos-López et al. 2019). In particular, selection for blackleg resistance is typically assessed from plant survivability against blackleg disease within an inoculated disease nursery (Marcroft et al. 2012; Potter et al. 2016). This survivability measure is calculated as the ratio of number of plants survived at physiological maturity relative to number of plants emerged and subsequently analysed using a univariate linear mixed model (LMM) (Fikere et al. 2018; Potter et al. 2007, 2016; Raman et al. 2020). Although this approach is intuitive, this simplified univariate LMM with survivability has several drawbacks. It fails to account for the correlation between emergence and mature plant counts (herein referred to as traits), nor does it consider the spatial trends and variations for each trait. Furthermore, observations with survivability greater than 100% (i.e., number of survived plants > emerged plants) have typically been thresholded to 100% as cited in Ganesalingam et al. (2013). Several factors may lead to these observations, including the emergence of more plants after the initial count and potential human error, such as under-counting at emergence or over-counting at maturity. By constraining these observations to 100%, this effectively disregards the true plant counts recorded in the field, potentially introducing bias into any subsequent analyses.

For multiple traits, bivariate or multivariate analyses have been shown to reduce selection bias and improve prediction accuracy in GS (Kadkol et al. 2021; Montesinos-López et al. 2016; Zhang-Biehn et al. 2021). Ganesalingam et al. (2013) recognised this and conducted a bivariate analysis of emergence and mature plant counts using LMMs, accounting for the between-trait correlation at the genetic and residual levels. They showed that the bivariate predictions were more accurate than univariate across all datasets. Nevertheless, there were several limitations in their analyses which form the objectives in this research. They assumed independence between genotypes, which may not be applicable in breeding programmes where genotypes are genetically related (Oakey et al. 2016). They also proposed selection measures for blackleg resistance that required a logarithmic transformation of the count data prior to analysis, which may not be easily generalisable to all datasets (O’Hara and Kotze 2010; Welham et al. 2014). In addition, Ganesalingam et al. (2013) highlighted a limitation with their residual variance structure, which included a three-way separable variance model to account for residual trait correlation and the separable spatial autocorrelation processes in the column and row directions. The separable nature of this structure is restrictive as it assumes the same spatial autocorrelation across both traits. Studies have stated the restrictiveness of this separability assumption, including multi-harvest data in sugarcane (Smith et al. 2007) and in perennial pasture (De Faveri et al. 2015). This assumption may not hold biologically as spatial trends may vary over time and across traits due to factors such as plant growth stage and soil moisture (De Faveri et al. 2023; Smith et al. 2007).

Given these limitations, this research aims to improve on the approach of Ganesalingam et al. (2013) in multiple aspects, to encourage the adoption of bivariate analyses for blackleg resistance in canola. To address the assumption of independence of genotypes, the incorporation of pedigree-based (numerator) or marker-based (genomic) relationship matrices would improve the model by considering the genetic relatedness between genotypes and henceforth borrowing strength from closely related relatives (Hayes et al. 2009; Piepho et al. 2008). This study incorporated a genomic relationship matrix that partitions total genetics into additive and non-additive components, enabling breeders to select for the best performing lines for parental crossing as well as commercial release in a single analysis (Oakey et al. 2006). In addition, marker effects can be generated to estimate the breeding values of genotyped but un-phenotyped genotypes (Meuwissen et al. 2001).

Additionally, we propose an alternative selection measure for blackleg resistance termed responsiveness (to blackleg disease), utilising the implicit random regression between emergence and mature counts in the bivariate model. Lemerle et al. (2006) first described this approach for incremental crop tolerance to weed competition, where tolerance of a genotype was defined as the deviation from the regression line between treatments, absence and presence of weeds. They asserted that this measure allows for the selection of competitive ability independent of the underlying yield potential. Similarly, “resistance” to blackleg disease can be defined as the ability of a genotype to maintain plant count in the presence of blackleg disease relative to its emergence count (prior to plant death from blackleg). Genotypes that are more responsive are expected to have greater survivability against blackleg and vice versa for less responsive genotypes. Studies have demonstrated the effectiveness of this selection approach for traits such as phosphorus use efficiency in barley (McDonald et al. 2018), drought tolerance (Dolferus et al. 2019), crown rot tolerance (Kelly et al. 2021) and heat stress adaptation (Telfer et al. 2021, 2022) in wheat.

To address the limitation of three-way separable residual models, De Faveri et al. (2017) developed a flexible model known as the two-dimensional invariant multivariate autoregressive of order 1 (2DIMVAR1). The novelty of 2DIMVAR1 is its ability to simultaneously model the correlation of residual effects between traits while allowing different spatial autocorrelation parameters for each trait, hence more suited for the repeated measurements of plant counts on the same plot (genotype) (De Faveri et al. 2017). By fitting this flexible 2DIMVAR1 model, studies have shown a significant improvement in model fit over the separable model in some cases (De Faveri et al. 2017, 2023; Keno et al. 2024), motivating the integration of 2DIMVAR1 residual model for our bivariate analyses.

To showcase these improvements, we analysed plant count data collected from canola blackleg nurseries across multiple environments within a commercial canola breeding programme managed by Australian Grain Technologies (AGT). This motivating example is illustrated in “Materials and methods”, along with the statistical methods to analyse them. To illustrate the key findings in this research and contrast them with historic approaches, the “Results” section is structured as follows: (1) univariate versus bivariate models; (2) bivariate baseline versus genomic marker models; (3) three-way separable versus 2DIMVAR1 residual models; (4) derivation of novel selection measure responsiveness; and (5) bivariate responsiveness versus historic univariate per cent survivability. Results show that our extended bivariate approach outperforms the historic bivariate analysis approaches. Major findings and their relevance in breeding programmes are discussed, and subsequently summarised in the “Conclusion” section. We believe that these results will encourage canola breeders to adopt this enhanced bivariate approach and potentially improve the genetic gain for blackleg resistance within their breeding programmes.

## Materials and methods

### Motivating data

The motivating dataset analysed in this study is comprised of advanced stage breeding trials conducted by Australian Grain Technologies’ (AGT) canola breeding programme. The trials were grown in Horsham (Victoria) and York (Western Australia) over 3 years, Roseworthy and Wanilla (South Australia) over 2 years, totalling ten environments (location by year combinations). Details of each trial are summarised in Table 1. All trials in 2021 and 2023 were sown as a partially replicated (*p*-rep) randomised design (Cullis et al. 2006) with 13 and 9 check varieties, respectively, while trials in 2022 were sown as a fully replicated randomised design with 8 check varieties. All trials were laid out in a rectangular array of plots indexed by columns and rows as shown in Table 1. Filler plots were added as necessary to ensure trials were laid out in rectangular array of plots. The trials were managed by AGT according to the best local practice at each location including herbicide application to control weeds. Seeding rate has been optimised based on average germination rate to encourage uniformity in plant emergence. For each blackleg disease nursery, genotypes were grown on blackleg stubble from the previous season, or inoculated with blackleg infested stubble, to promote high disease pressure. The time of sowing was also delayed to further encourage the establishment of blackleg disease in the nursery.

The traits of interest in this paper are emergence and mature plant counts of canola, which are used to derive per cent survival rate (BLsur), using methods set out by Potter et al. (2007). BLsur is calculated by dividing number of plants survived at maturity (BLmat) by number of plants at emergence (BLeme) and subsequently transforming it to percentage. For each plot, the total number of seedlings emerged were counted at open cotyledon stage and subsequently recounted at physiological maturity to determine the number of plants survived. The timing of BLeme is therefore crucial in establishing an accurate baseline count after emergence but before plant death occurs (Potter et al. 2007). A summary of the phenotype data is presented in Table 1.

### Marker data

Marker genotyping was performed using a custom Axiom™ Affymetrix array containing 9,464 single nucleotide polymorphism (SNP) markers. For each marker, individual genotypes were coded as either −1 (homozygous minor allele), 0 (heterozygous) or 1 (homozygous major allele). Therefore, the complete marker matrix is a matrix of *m* genotypes by 9,464 markers. Prior to the construction of genomic relationship matrix ($\varvec{G}$), markers with minor allele frequency < 3%, missing values frequency > 20% and heterozygosity > 40% were excluded, leaving 8,179 markers remaining. Missing marker data were imputed using marker means (Montesinos-López et al. 2016; Rutkoski et al. 2016) of lines from within each breeding cohort. The matrix $\varvec{G}$ was then constructed with the R package *pedicure* (Butler 2019) from the remaining marker matrix ($\varvec{M}$), subsequently scaled by $\varvec{G}_s = \varvec{MM}^T/ r$, where $r= trace(\varvec{G}) / m$ (Forni et al. 2011; Norman et al. 2017).

**Table 1:** Summary of blackleg nursery trials from 2021 to 2023 at four locations across western and southern Australia

								Mean ± SD		
Dataset	Site	Year	Geno	Cols	Rows	Format	Plot size^a^	BLsur (%)	BLeme	BLmat
HS21	Horsham	2021	240	12	36	6 row plot	1.5 x 5.0	67.9 ± 20.0	81.2 ± 24.9	55.1 ± 21.9
YK21	York	2021	240	12	36	3 row plot	0.6 x 3.0	75.5 ± 16.3	67.6 ± 18.9	51.3 ± 18.1
WN22	Wanilla	2022	82	6	33	single row	10.0	51.1 ± 23.5	75.7 ± 27.8	39.0 ± 23.3
HS22	Horsham	2022	82	12	16	6 row plot	1.5 x 5.0	53.5 ± 22.0	80.5 ± 26.5	43.1 ± 24.1
RS22	Roseworthy	2022	82	32	6	single row	3.5	39.5 ± 23.7	22.9 ± 12.6	9.5 ± 7.7
YK22	York	2022	82	12	16	3 row plot	0.6 x 3.0	81.4 ± 12.0	59.6 ± 17.9	48.4 ± 15.0
WN23	Wanilla	2023	327	12	30	single row	5.0	38.8 ± 20.2	36.0 ± 11.9	14.1 ± 8.9
HS23	Horsham	2023	327	12	30	6 row plot	1.2 x 4.0	36.7 ± 21.8	41.7 ± 13.0	15.2 ± 11.6
RS23	Roseworthy	2023	327	20	20	single row	4.0	77.5 ± 14.7	86.0 ± 16.0	66.4 ± 16.3
YK23	York	2023	327	12	30	3 row plot	0.6 x 3.0	56.8 ± 17.6	83.1 ± 19.3	47.1 ± 18.4

^a^Plot size shown in metres, width x length for plot format, length for single row format

Geno = Genotypes; Cols = Columns; SD = Standard Deviation

### Statistical methods

#### Univariate modelling

The count data collected for blackleg survival, BLeme and BLmat, constitute the two traits for each genotype. The data are arranged as rows within columns within traits, and the baseline univariate model was used to properly identify the appropriate spatial models for each trait independently. The identification of spatial models that appropriately account for the spatial dependency between neighbouring plots and trends across the trial area will improve accuracy of predicted genetic effects (Gilmour et al. 1997; Gogel et al. 2018). The univariate model specified in this section acts as a baseline model for comparison with the bivariate model specified in the proceeding sections. Let *m* be the number of genotypes, *r* and *c* be the number of rows and columns, respectively. The trial is laid out in a rectangular array of *r* rows and *c* columns, such that the total number of plots is given by $n=rc$. The initial baseline univariate linear mixed model for either trait had the form

1 $$\begin{aligned} \varvec{y} = \varvec{X\tau } + \varvec{Z}_u\varvec{u} + \varvec{Z}_g\varvec{g} + \varvec{e} \end{aligned}$$

where $\varvec{y}$ is the $(n \times 1)$ vector of observed responses for each trait. $\varvec{\tau }$ is a vector of *t* fixed effects with associated $(n \times t)$ design matrix $\varvec{X}$. The vector $\varvec{\tau }$ contains the overall site mean for all genotypes and may also include the linear regression of row and/or column numbers (as necessary) to account for global trends across the experiment (Gilmour et al. 1997). $\varvec{u}$ is the vector of *b* non-genetic random effects associated with the design of the experiment such as rows and columns (as necessary), with corresponding $(n \times b)$ design matrix $\varvec{Z}_u$. $\varvec{g}$ is the vector of total genetic random effects of *m* genotypes with its corresponding $(n \times m)$ design matrix $\varvec{Z}_g$ that relates the count data obtained from plots to corresponding genotypes. The genetic effects are assumed to have distribution $\varvec{g} \sim N(0,\sigma _g^2 \varvec{I}_m)$, where $\sigma _g^2$ represent the variance of the genetic effects for a given trait and $\varvec{I}_m$ is an identity matrix assuming independence between *m* genotypes.

Any other sources of non-genetic variation attributable to the environment were modelled through the residual error $\varvec{e}$. As advocated by Gilmour et al. (1997), a two-dimensional separable autoregressive process of order one $(AR1 \otimes AR1)$ was used in the column and row directions of the experiment, where the variance of residual error $\varvec{e}$ was assumed to have the following form:

$$\begin{aligned} \text {var}(\varvec{e}) = \varvec{R} = \varvec{\Sigma }_c \otimes \varvec{\Sigma }_r \end{aligned}$$

where $\varvec{e} \sim N(0,\sigma ^2 \varvec{R})$, $\varvec{\Sigma }_c$ and $\varvec{\Sigma }_r$ are the first-order autoregressive correlation matrices for the column and row directions, respectively.

#### Bivariate modelling

To extend model 1 to a bivariate approach, let the response variable $\varvec{y} = (\varvec{y}_1^T,\varvec{y}_2^T )^T$to be a $(2n \times 1)$ vector of observed responses for the two traits, BLeme and BLmat, measured on *n* plots. A bivariate LMM was used to model $\varvec{y}$ in the form

2 $$\begin{aligned} \varvec{y} = (\varvec{X} \otimes \varvec{I}_2) \varvec{\tau } + (\varvec{Z}_u \otimes \varvec{I}_2)\varvec{u} + (\varvec{Z}_g \otimes \varvec{I}_2)\varvec{g} + \varvec{e} \end{aligned}$$

where $\varvec{\tau }$ is a vector of *t* fixed effects with associated $(2n \times t)$ design matrix $\varvec{X}$. Analogous to the univariate model, the vector $\varvec{\tau }$ contains the overall site mean of all genotypes for each trait and may also include any global spatial trends as identified in the univariate model 1. $\varvec{u}$ is the vector of *b* non-genetic random effects identified in the univariate analyses for each trait, with corresponding $(2n \times b)$ design matrix $\varvec{Z}_u$. To model the vector of random total genetic effects for each of the traits, $\varvec{g} = (\varvec{g}_1, \varvec{g}_2 )$, we assume a distribution for the genetic effects in the form

$$\begin{aligned} \varvec{g} \sim N(0,\varvec{\Sigma }_g \otimes \varvec{I}_m) \text { where } \varvec{\Sigma }_{g} = \begin{bmatrix} \sigma _{g_1}^2 & \rho _{g_1 g_2} \\ \rho _{g_1 g_2} & \sigma _{g_2}^2 \end{bmatrix} \end{aligned}$$

and $\varvec{\Sigma }_{g}$ represents a $2 \times 2$ fully parameterised unstructured correlation matrix with heterogenous variances on the diagonal. This parameterisation ensures we have a separate genetic variance for each trait with a genetic correlation in the off-diagonals to model the genetic connectivity between traits. As advocated by Oakey et al. (2006), the random total genetic effects $\varvec{g}$ can be partitioned into vectors of additive $(\varvec{g}_a)$ and non-additive $(\varvec{g}_p)$ genetic effects. The genetic effects in model 2 can now be extended to take the form:

3 $$\begin{aligned} \varvec{g} = \varvec{g}_a + \varvec{g}_p \end{aligned}$$

where the additive genetic effects $\varvec{g}_a$ are assumed to be distributed in the form, $\varvec{g}_a \sim N(0,\varvec{\Sigma }_a \otimes \varvec{G}_s)$ with $\varvec{G}_s$ as a genomic relationship matrix previously defined. The non-additive genetic effects $\varvec{g}_p$ are assumed to be distributed in the form, $\varvec{g}_p \sim N(0,\varvec{\Sigma }_p \otimes \varvec{I}_m )$. The random genetic effects, $\varvec{g}_a$ and $\varvec{g}_p$, of each trait were mapped accordingly to the corresponding genotypes in the dataset with the $(2n \times m)$ design matrix $\varvec{Z}_g$. It is important to note that $\varvec{g}_a$ and $\varvec{g}_p$ are assumed to be mutually independent, such that the vector of total genetic effects $\varvec{g}$ for *m* genotypes are now assumed to be distributed as $\varvec{g} \sim N(0,\varvec{\Sigma }_a \otimes \varvec{G}_s + \varvec{\Sigma }_p \otimes \varvec{I}_m )$. $\varvec{\Sigma }_a$ and $\varvec{\Sigma }_p$ are both $2 \times 2$ matrices analogous to $\varvec{\Sigma }_g$ but now for the additive and non-additive genetics respectively.

To model the residual error $\varvec{e}$, two methods were explored such that the residual covariance matrix considers the spatial autocorrelation and genetic correlation between each trait. Here, local spatial trends were modelled with a separable autoregressive process of order 1 $(AR1 \otimes AR1)$ in the row and column directions as advocated by Gilmour et al. (1997).

1. The first approach utilises a three-way (trait by column by row) separable variance-covariance structure as advocated by Ganesalingam et al. (2013) which is assumed to be:
  $$\begin{aligned} \text {var}(\varvec{e}) = {\varvec{\Sigma }_e} \otimes \varvec{\Sigma }_c \otimes \varvec{\Sigma }_r \end{aligned}$$
  where $${\varvec{\Sigma }_e} = \begin{bmatrix} \sigma _{e_1}^2 & \rho _{e_1 e_2} \\ \rho _{e_1 e_2} & \sigma _{e_2}^2 \end{bmatrix}$$ is a $2 \times 2$ fully parameterised unstructured correlation matrix with heterogenous variances on the diagonal, representing the residual error variance for each trait. The off-diagonals are the correlation between the residual errors which accounts for the repeated measurement nature of the data where each plot is counted at two sampling times. $\varvec{\Sigma }_c$ and $\varvec{\Sigma }_r$ are spatial autocorrelation matrices for the column and row trends, respectively. This three-way separable structure, however, assumes a common spatial correlation for each sampling time, and is therefore restrictive and unlikely to hold in a biological context.
2. As described by De Faveri et al. (2017), a more flexible approach is to model the residual error effects with a two-directional invariant multivariate autoregressive of order 1 (2DIMVAR1) model. The derivation and underlying theory of 2DIMVAR1 is described in detail by De Faveri et al. (2017). The variance structure for the 2DIMVAR1 model in a bivariate context can be written as follows:
  $$\begin{aligned} \text {var}(\varvec{e}) = \sum _{s = 1}^2 \varvec{\Sigma }_{cs} \otimes \varvec{\Sigma }_{rs} \otimes \varvec{p}_s \varvec{p}_s^T \end{aligned}$$
  where $\varvec{\Sigma }_{cs}$ and $\varvec{\Sigma }_{rs}$ are the correlation matrices for the first-order autoregressive processes for columns and rows, respectively, and $\varvec{p}_s$ is a reduced rank matrix, which can be thought of as a special case of a factor analytic model with specified variances constrained to zero (De Faveri et al. 2017).

#### Computational modelling

The analysis of blackleg survival data was conducted using LMMs with the estimation of variance parameters by residual maximum likelihood (REML) (Patterson and Thompson 1971). Given these REML estimates, empirical best linear unbiased predictions (EBLUPs) of the random effects can be obtained. All LMMs were fitted using the flexible *ASReml-R* package (Butler et al. 2017) available in the R statistical computing environment (R Core Team 2025). To perform diagnostics on the models fitted, a sample variogram of the residuals was plotted to determine the appropriate spatial models and residual plots to assess model assumptions (Gilmour et al. 1997). Through these model diagnostics, potential outliers in the data were identified and therefore informed if any additional fixed and/or random terms were necessary in the model. To meet the assumption of normality and homogeneity of variance, a square-root transformation was applied as necessary. For all marker models, we incorporated a genomic relationship matrix that was constructed from post-filtered marker data using the *pedicure* R package (Butler 2019).

To determine the most parsimonious model for each dataset between nested models, the Akaike Information Criterion (AIC) (Akaike 1998) and REML log-likelihood ratio test (REMLRT) were used. Models with significant *p*-values $(<0.05)$ from REMLRT and smaller AIC values are more parsimonious and provide a better fit to the data.

#### Heritability and accuracy

Heritability can be defined as the squared correlation between the true and predicted genetic effects (Falconer and Mackay 1996). This definition has therefore allowed for heritability to be thought of as analogous to reliability (square of accuracy) as defined by Mrode and Thompson (2005). Given this definition and the complexity of our bivariate models, we used the generalised heritability defined in Oakey et al. (2006) given by

4 $$\begin{aligned} \varvec{H}^2 = \left( 1 - \frac{\text {tr}(\varvec{G}^{-1} \varvec{C}^{ZZ})}{m}\right) \end{aligned}$$

where $\varvec{G}$ is the variance of the random genetic effects, $\varvec{C}^{ZZ}$ is the prediction error variance (PEV) matrix for the predicted genetic effects and *m* is the number of genotypes. Using model 3, the broad-sense heritability $(\varvec{H}^2)$ of each trait can be calculated by considering $\varvec{G} = \sigma _a^2 \varvec{G}_s + \sigma _r^2 \varvec{I}_m$ and narrow sense heritability $(\varvec{h}^2)$ by considering $\varvec{G} = \sigma _a^2 \varvec{G}_s$. Subsequently, prediction accuracies for the additive and total genetic effects can be immediately calculated by taking the square root of $\varvec{h}^2$ and $\varvec{H}^2$, respectively.

#### Regression of genetic effects from bivariate model

In this section, we propose a method to investigate blackleg resistance across the genotypes by considering the random regression implicit in the bivariate correlation structure of the genetic effects (Lemerle et al. 2006). In this context, the approach can be viewed as a random regression of the EBLUPs for BLmat against BLeme. In model 3, the partitioning of total genetic effects into additive and non-additive genetics means we can conduct a separate regression for each set of effects. For brevity, we focus on the additive genetic effects, $\varvec{g}_a$.

The two sets of genetic effects, namely $\varvec{g}_{a_1}$ for BLeme and $\varvec{g}_{a_2}$ for BLmat, are described in the form of a regression where $\varvec{g}_{a_2}$ against $\varvec{g}_{a_1}$ can be written as

5 $$\begin{aligned} \varvec{g}_{a_2} = \beta _a\varvec{g}_{a_1} + \varvec{g}_{a_r} \end{aligned}$$

where the slope of the regression, $\beta _a = \sigma _{a_{12}}/\sigma _{a_1}^2$. From this regression between BLeme and BLmat, two key parameters can be derived to form the basis of the new selection measure for blackleg resistance: (1) the slope of the regression, $\beta _a$ is an estimate of the average survivability against blackleg across all genotypes; (2) the deviation (residuals) from the regression line, $\varvec{g}_{a_r}$, is an estimate of the genetic deviation of each genotype from the average survivability. The genetic deviation therefore constitutes a new measure for selection, herein referred to as responsiveness to blackleg disease (BLresp).

## Results

### Univariate versus bivariate

In multi-trait datasets such as the blackleg survival counts, it is important to first analyse the traits independently to identify sources of spatial trends and potential outliers before implementing a more complex model. In our datasets, we found that the phenotypic correlation ($r_{raw}$) between BLeme and BLmat ranged from 0.50 (HS23) to 0.83 (YK22) with a mean of 0.64, showing a moderate to strong correlation between traits (Table 2). This suggested that a bivariate approach may be more suitable for the analysis of the two traits as their genetic and residual effects may also be correlated.

Hence, we fitted a bivariate model and found that it was a better fit over the univariate approach across all datasets, as demonstrated by the lower AIC values (Table 2). This can be largely attributed to the bivariate structure that considers the genetic correlation between traits and the repeated measurement nature in the residual errors. The between-trait correlations of total genetic effects ($\rho _g$) and residual error effects ($\rho _e$) were subsequently extracted from the bivariate models. As anticipated, a moderate to strong genetic correlation was observed across all sites, ranging between 0.49 (YK23) and 0.91 (YK22) (Table 2). The error correlations ranged between 0.32 (HS23) and 0.81 (HS21), highlighting the variability in temporal correlation between repeated measurements of the same plot.

In addition, the mean prediction accuracy of the EBLUPs for genotypes at a given dataset was calculated for both traits and compared between models in Table 2. Here, accuracies were largely similar between the univariate and bivariate models for both traits. Contrary to anticipation, prediction accuracies of the genetic effects for BLeme were generally similar to BLmat, as BLeme has typically been considered to be more environmentally driven (Kirkegaard et al. 2021).

**Table 2:** Summary of prediction accuracies of the EBLUPs for BLeme and BLmat, AIC values for univariate and bivariate models, and between-trait correlations of total genetic effects $(\rho _g)$, residual errors $(\rho _e)$, and raw phenotypic data $(r_{raw})$

	Univariate			Bivariate					
	Accuracy		AIC	Accuracy		AIC	$\rho _g$	$\rho _e$	$r_{raw}$
Dataset	BLeme	BLmat		BLeme	BLmat				
HS21	0.66	0.86	5917	0.63	0.89	5629	0.57	0.81	0.72
YK21	0.89	0.86	5600	0.89	0.87	5204	0.85	0.72	0.80
WN22	0.94	0.90	2660	0.95	0.91	2599	0.75	0.34	0.55
HS22	0.94	0.96	2601	0.94	0.96	2533	0.62	0.54	0.61
RS22	0.89	0.88	486	0.89	0.90	425	0.74	0.56	0.64
YK22	0.95	0.95	2289	0.96	0.96	2114	0.91	0.68	0.83
WN23	0.49	0.52	806	0.50	0.52	692	0.69	0.50	0.53
HS23	0.67	0.92	824	0.74	0.93	743	0.62	0.32	0.50
RS23	0.86	0.83	4478	0.86	0.82	4238	0.74	0.69	0.68
YK23	0.67	0.82	4584	0.67	0.84	4503	0.49	0.50	0.58

### Bivariate baseline versus genomic

**Table 3:** Summary of comparisons between bivariate baseline and genomic marker models including total genetic variance ($\sigma ^2_g$), additive ($\sigma ^2_a$) and residual genetic ($\sigma ^2_r$) variances, per cent of additive variance (Add. (%)) for each trait and residual log-likelihood (LL) values of each model

	Bivariate					Bivariate genomic										
	BLeme		BLmat			BLeme					BLmat					
Dataset	$\sigma _{g}^{2}$	$H^2$	$\sigma _{g}^{2}$	$H^2$	LL	$\sigma _{a}^{2}$	$\sigma _{r}^{2}$	Add. (%)^a^	$H^2$	$h^2$	$\sigma _{a}^{2}$	$\sigma _{r}^{2}$	Add. (%)^a^	$H^2$	$h^2$	LL
HS21	98.6	0.39	235.0	0.79	$-$2800	*b*	97.2	0	0.38	0.00	153.9	135.1	53	0.74	0.27	$-$2782
YK21	248.3	0.80	208.9	0.76	$-$2588	*b*	247.0	0	0.79	0.00	73.8	154.9	32	0.76	0.29	$-$2565
WN22	543.6	0.89	300.1	0.83	$-$1289	*b*	543.8	0	0.89	0.00	206.0	138.9	60	0.83	0.54	$-$1277
HS22	528.6	0.89	495.2	0.93	$-$1252	*b*	529.3	0	0.89	0.00	343.5	267.1	56	0.91	0.48	$-$1245
RS22	1.0	0.79	1.2	0.80	$-$198	*b*	1.0	0	0.79	0.00	0.4	0.8	32	0.79	0.26	$-$195
YK22	256.8	0.91	184.8	0.91	$-$1040	*b*	256.8	0	0.91	0.00	*b*	184.8	0	0.91	0.00	$-$1040
WN23	0.2	0.25	0.4	0.27	$-$336	0.3	0.0	90	0.21	0.18	1.2	0.0	100	0.24	0.24	$-$312
HS23	0.4	0.54	1.3	0.86	$-$357	0.3	0.3	49	0.49	0.44	2.4	0.3	89	0.76	0.44	$-$299
RS23	118.2	0.74	119.9	0.67	$-$2099	121.2	59.9	67	0.71	0.24	170.9	51.1	77	0.64	0.27	$-$2063
YK23	74.2	0.45	174.8	0.70	$-$2236	117.7	18.8	86	0.34	0.25	311.9	0.0	100	0.39	0.39	$-$2164

^a^Per cent of additive genetic variance ($\sigma _{a}^{2}$) of the total genetic variance ($\sigma _{a}^{2}$ + $\sigma _{r}^{2}$)

^b^additive genetic variance component not significant (REMLRT of $H_{o}$, $\sigma _{a}^{2}$ = 0)

To extend the bivariate baseline model to incorporate genetic marker information, a genomic relationship matrix was integrated to consider the genetic relatedness between genotypes, herein referred to as the bivariate genomic model. A heatmap of this genetic relatedness between genotypes is presented in Fig. S1 (Online Resource 1), indicating a diverse range of genotypes in the data analysed. As the genomic model partitioned total genetic effects into additive and non-additive (residual) components, total genetic variance can be acquired through the sum of the two variances ($\sigma ^2_a + \sigma ^2_r$). The genetic variances, narrow and broad sense heritabilities of each trait, and residual log-likelihood values of the two models were contrasted in Table 3.

The consideration of genetic relatedness improved model fit across all datasets as reflected by the higher LL values. The only exception was YK22 where the additive components for both traits were not significant. For datasets with significant additive component (REMLRT, $p < 0.05$), total genetic variances for BLeme and BLmat were consistently higher in the genomic model, increasing by an average of 28% and 55%, respectively, over the baseline model. This suggests that the inclusion of genetic marker information enabled the model to account for more genetic variance by borrowing information from closely related genotypes. BLmat was also shown to benefit more than BLeme from the inclusion of markers, as every dataset except YK22 had a significant additive component and a higher proportion of additive genetic variance (Add. %). This was also reflected in $h^2$ and $H^2$ as BLmat was generally more heritable than BLeme. Lastly, note that the $H^2$ for genomic models were marginally lower than the baseline model, which is likely due to the upward bias of an incorrect baseline model that assumed independence between genotypes (Costa e Silva et al. 2004; Oakey et al. 2006).

### Residual models

Table 4 contrasts the modelling of residual error effects with 2DIMVAR1 against the historic three-way separable model. For all datasets, an increase in LL value indicated that the 2DIMVAR1 residual model was a better fit to the data. Notably, 2DIMVAR1 was a significant improvement over the separable model (REMLRT, $p < 0.05$) in five of ten datasets. The use of 2DIMVAR1 allowed the bivariate model to consider between-trait temporal correlation as well as different spatial correlation parameters for each measurement time (trait) at the residual level, providing a better fit for survival count data. Furthermore, assuming the same spatial autocorrelation parameters across traits with the separable model can be restrictive as demonstrated in Table 4. The estimated spatial autocorrelation values from 2DIMVAR1 showed that spatial trends may vary substantially across traits. For instance, in RS22, 2DIMVAR1 estimated the spatial autocorrelation in the column direction for BLeme and BLmat to be 0.434 and 0.256, respectively, whereas the separable model assumed the same autocorrelation value (0.249) for both traits. The presence of strong local spatial trends was observed at sites with high spatial autocorrelation such as RS23 ($\phi _r$ = 0.839). On average, there was a stronger spatial trend in the row direction than the column direction across the ten datasets.

For datasets where 2DIMVAR1 was significant over the separable model, the improved modelling of residual effects may directly affect the prediction of genetic effects for each trait (De Faveri et al. 2017; Verbyla et al. 2021), ultimately resulting in changes in genotype rankings for selection. Here, the additive and non-additive genetic correlations for the separable and 2DIMVAR1 residual models were generally similar across all datasets; hence, they were omitted from Table 4. This finding suggested that there may only be minimal re-ranking of genotypes between the two residual models. As shown in Fig. S2 (Online Resource 3), although there were differences in predicted genetic effects between the separable and 2DIMVAR1 residual models, the top 20% of ranked genotypes were largely similar and thus may not result in a significant impact on breeding selection decisions in our datasets.

**Table 4:** Summary of bivariate models fitted with three-way separable and 2DIMVAR1 residual models, spatial autocorrelation for column ($\phi _c$) and row ($\phi _r$) directions, residual log-likelihood (LL), and *p*-values of REML log-likelihood ratio test (REMLRT)

	Separable			2DIMVAR1					
				BLeme		BLmat			
Dataset	$\phi _c$	$\phi _r$	LL	$\phi _c$	$\phi _r$	$\phi _c$	$\phi _r$	LL	REMLRT
HS21	0.141	0.369	$-$2782	0.140	0.505	0.149	0.540	$-$2776	0.002 **
YK21	0.249	0.378	$-$2565	0.350	0.312	0.227	0.381	$-$2563	0.084 n.s
WN22	0.010	0.342	$-$1277	0.098	0.472	$-$0.052	0.183	$-$1274	0.064 n.s
HS22	0.193	0.127	$-$1245	0.222	0.116	0.216	$-$0.185	$-$1242	0.039 *
RS22	0.249	0.217	$-$195	0.434	0.263	0.256	0.213	$-$192	0.031 *
YK22	$-$0.067	0.061	$-$1040	$-$0.061	$-$0.027	$-$0.061	$-$0.037	$-$1039	0.307 n.s
WN23	0.029	$-$0.030	$-$312	0.135	$-$0.171	0.002	0.024	$-$306	0.002 **
HS23	0.238	0.155	$-$299	0.180	0.123	0.072	0.054	$-$298	0.240 n.s
RS23	$-$0.081	0.740	$-$2063	$-$0.151	0.839	$-$0.077	0.695	$-$2061	0.041 *
YK23	0.051	0.087	$-$2164	0.178	0.098	$-$0.077	0.086	$-$2162	0.104 n.s

### Responsiveness

Leveraging the random regression implicit in the bivariate correlation structure, we defined a third trait, blackleg responsiveness (BLresp), to select for blackleg resistance (see Materials and methods). A key regression parameter is its slope ($\beta$), which could be interpreted as the average survivability of genotypes against blackleg at a given site. For instance, in RS23, the regression slope for additive EBLUPs ($\beta _a$) was estimated at 0.88 (Table S1, Online Resource 2). This suggests that, on average, for every one plant increase at BLeme, 0.88 plant survived at BLmat post-blackleg infection, translating to an average survival rate of 88%.

**Fig. 1:**
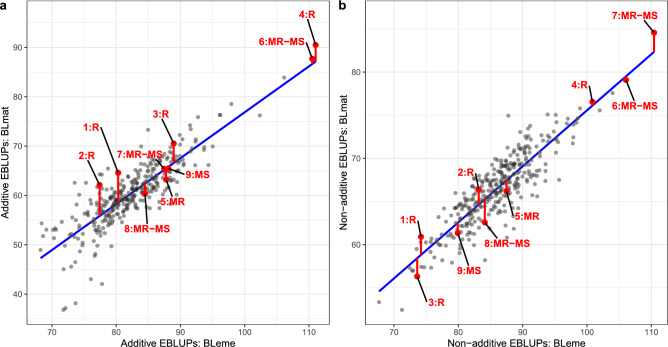
The derivation of BLresp in dataset RS23. The additive (**a**) and non-additive (**b**) EBLUPs of the mature counts (BLmat) against the emergence counts (BLeme), with the regression line plotted in blue. Labelled in red are the vertical deviations of check varieties from the regression line along with their NVT blackleg ratings (GRDC 2023). Check varieties and their blackleg ratings are summarised in Table 5

To visualise the regression for RS23, we used the additive and non-additive EBLUPs from the bivariate genomic model to demonstrate the derivation of BLresp (Fig. 1). The REML estimates of additive and non-additive genetic correlations between BLeme and BLmat for RS23 were 0.77 and 0.79, respectively (Table S1, Online Resource 2). Although there was general agreement between the two sets of genetic effects, this indicated some genotype re-rankings from BLeme to BLmat, suggesting differential responses (resistance) among genotypes to blackleg. This was evident in Fig. 1a, b where vertical deviations from the regression line represent the BLresp of individual genotypes. To interpret this new measure, a large positive deviation (above regression line) indicates that a genotype is expected to perform above average for resistance in the presence of blackleg, and vice versa for negative deviations (below regression line). The differences in magnitude and direction of these vertical deviations indicated a diversity in blackleg resistance among genotypes.

**Fig. 2:**
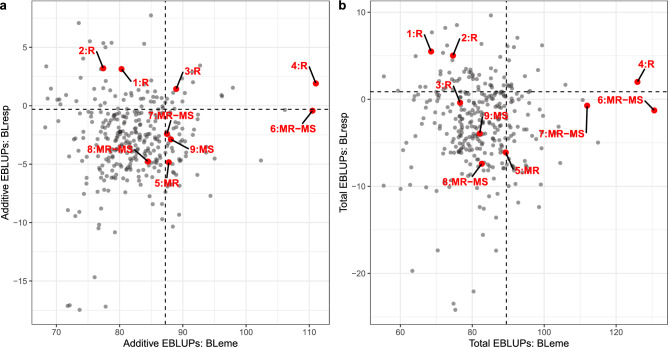
The additive (**a**) and total (additive + non-additive) (**b**) EBLUPs of BLresp against BLeme. The vertical-dotted line indicates the cut-off for top 20% of genotypes for BLeme while the horizontal line represents the cut-off for top 20% of genotypes for BLresp. Labelled in red are check varieties and their corresponding NVT blackleg ratings (GRDC 2023). Check varieties and their blackleg ratings are summarised in Table 5

Figures 2a, b demonstrated the relationship between the EBLUPs of BLresp and BLeme for additive and total genetic effects, respectively. These figures clearly demonstrated that BLresp is statistically independent of BLeme, allowing for selection of genotypes with superior blackleg resistance independent of their underlying BLeme. Furthermore, the rankings from BLresp EBLUPs of check varieties largely agreed with the independent blackleg resistance ratings published by the Australian National Variety Trials (NVT) programme in 2023 (GRDC 2023) presented in Table 5. This is evident in Fig. 2a, b, where varieties 1 to 4 with “R” ratings generally ranked in the top 20% of population followed by varieties 6 to 9 with “MR-MS” and “MS” ratings. Only one variety appeared to be misaligned relative to other check varieties in this particular dataset, given its “MR” rating.

**Table 5:** Summary of check varieties and their 2023 NVT blackleg ratings (bare seed) (GRDC 2023)

Variety	Variety name	Blackleg rating
1	HyTTec Trident	R
2	HyTTec Trifecta	R
3	HyTTec Trophy	R
4	DG Bidgee TT	R
5	Renegade TT	MR
6	ATR-Stingray	MR-MS
7	ATR-Wahoo	MR-MS
8	Bandit TT	MR-MS
9	ATR-Bonito	MS

Ratings definition: Resistant (R); Resistant to Moderately Resistant (R-MR); Moderately Resistant (MR); Moderately Resistant to Moderately Susceptible (MR-MS); Moderately Susceptible (MS)

### Comparison of selection measures

**Fig. 3:**
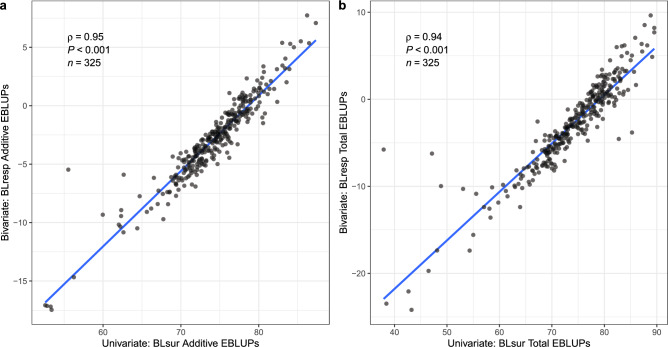
The additive (**a**) and total (**b**) EBLUPs of BLresp against the historic BLsur in RS23. $\rho$ represents the Spearman’s rank correlation coefficient between the two selection measures while *P*-value shows the significance of the linear regression between BLresp and BLsur (blue line). *n* represents the number of genotypes in RS23

To compare BLresp as a novel selection measure for blackleg resistance against the historic per cent survival (BLsur), two methods were employed to highlight discrepancies in genotype rankings and henceforth selection decisions between the two measures. Firstly, the additive and total genetic EBLUPs of BLresp were plotted against BLsur to visualise their relationship as presented in Fig. 3a, b. The Spearman rank correlation ($\rho$) was used to measure the association of genotype rankings between the two measures. Although the two selection measures were highly correlated for dataset RS23, there were differences in genetic effects particularly at the bottom half of the genotypes that may have been incorrectly ranked using BLsur. In the context of early generation breeding lines, the incorrect ranking of genotypes would have led breeders to mistakenly cull these lower-ranked genotypes, thus losing genetic materials due to a less robust selection approach.

The second method calculates the percentage of mismatched genotypes to investigate the re-ranking of genotypes when using BLsur over BLresp. This is done by first listing genotypes in the top and bottom 20% for each selection measure, subsequently comparing the absence of genotypes between lists. For example, in the top 20% of genotypes at RS23, the mismatched genotypes between BLsur and BLresp were 20.0% and 29.2% for the additive and total EBLUPs, respectively. In the bottom 20%, the mismatched genotypes were 18.5% and 16.9%, respectively. These discrepancies between lists have significant implications towards breeders’ selection decisions to advance or cull breeding lines. This could potentially result in lines with poor blackleg resistance being carried forward in the breeding programme while lines with greater resistance being culled. Beyond these simple assertions, further investigation of practical consequences of genotype misclassification would require a thorough simulation which is a subject of further research.

## Discussion

The results presented in this study optimised the bivariate analysis for canola survivability against blackleg disease by addressing the shortcomings of historic univariate and bivariate approaches. The historic univariate analysis of per cent survivability omits the consideration of correlation between traits BLeme and BLmat as well as their individual spatial trends and residual errors (Ganesalingam et al. 2013). The importance of spatial modelling is well established in the literature as shown by Gilmour et al. (1997) and Smith et al. (2001), demonstrating a reduction in effective error variance and improvement in prediction accuracy. A univariate analysis of each trait independently using model 1 can model their spatial variation separately but fails to consider the genetic and residual error correlations between them. Therefore, Ganesalingam et al. (2013) proposed model 2 with a three-way separable residual model that adequately modelled the between-trait correlations at the genetic and residual levels while considering their spatial trends. For comparison, we replicated their model with our datasets and validated that the bivariate model was indeed a more robust model than the univariate analysis of each trait across all datasets. Subsequently, we integrated three innovative solutions that addressed the limitations in their bivariate model, where we found that the integration of genomic markers, a flexible 2DIMVAR1 residual model and a novel selection measure BLresp provided a more comprehensive analysis for blackleg resistance in variety selection trials.

To extend the bivariate approach by Ganesalingam et al. (2013) to use genomic marker information, we incorporated a genomic relationship matrix to more readily reflect the genetic relatedness between genotypes in a breeding programme. Importantly, this also enables breeders to select genotypes with superior blackleg resistance for both parental crossing and commercial release in a single analysis (Oakey et al. 2006). While we found that the inclusion of genomic markers was significant at more datasets for BLmat compared to BLeme, the total genetic variance for these traits was highly comparable and in some cases BLeme actually had a greater genetic variance. This could be due to the highly polygenic nature of canola seed germination and vigour (Hatzig et al. 2015) which may lead to complex epistatic effects (Hill 2010; Visscher et al. 2008). Another plausible reason is that genomic markers included in the present study have not captured SNPs that could explain variations associated with seedling emergence.

We also explored a more flexible 2DIMVAR1 model for the modelling of residual errors between traits. In many cases, fitting the 2DIMVAR1 residual model provided an extra flexibility such that it allows for differing spatial correlation for each trait, thus making more sense biologically. Although our results found that 2DIMVAR1 significantly improved model fit over the separable model in several datasets, there was little impact on their genotype rankings as reflected by the minimal changes in genetic correlations. Nevertheless, it is important to note that studies by De Faveri et al. (2017, 2023) and Verbyla et al. (2021) have demonstrated that the improvement in modelling of residual error effects can have an impact on the predicted genetic effects which ultimately influence genotype selection. As the number of traits or measurement times increase, De Faveri et al. (2023) acknowledged the computational difficulties of fitting the 2DIMVAR1 residual model. Therefore, it may be desirable to explore other alternatives for spatio-temporal modelling, including reduced rank tensor smoothing splines (Verbyla et al. 2021), tensor P-splines (Pérez-Valencia et al. 2024), and other spatio-temporal approaches in a Bayesian context (Lindgren and Rue 2015; Selle et al. 2019) implemented in packages such as R-INLA (Rue et al. 2009) that utilised sparse model structures to reduce computational complexity. Hence, it is still encouraged to explore flexible residual models when possible for multi-trait, multi-harvest or multi-treatment datasets to more adequately model their multivariate residual error effects over space and time to obtain more accurate predictions for genetic effects.

Lastly, we addressed the constrained selection measures in Ganesalingam et al. (2013) by proposing a selection measure that can be readily derived from the bivariate correlation structure. The BLresp trait enables breeders to select for genotypes that demonstrate greater than expected BLmat given their underlying BLeme, thus reflecting the ability of a genotype to maintain plant count under blackleg disease pressure. The differential responses among genotypes to blackleg disease allow the ranking and selection of blackleg resistance using the BLresp EBLUPs. In this study, we found that the novel selection measure BLresp consistently ranked check varieties that were largely in agreement with their corresponding NVT blackleg resistance ratings, showing that BLresp can serve as a useful summary of the predicted genetic effects from the bivariate analysis. Furthermore, the methods in the present study for the derivation of BLresp from the bivariate genetic regression as a selection measure can be easily generalisable to other biotic and abiotic tolerance or resistance studies such as crown rot disease and drought stress.

In host-pathogen relationships such as canola and blackleg, breeders are generally interested in incorporating both quantitative resistance (QR) (or adult plant resistance) and major gene resistance (MGR) in canola varieties to promote the durability of blackleg resistance (Brun et al. 2010; Delourme et al. 2014). MGR is typically present from the seedling stage that provides complete resistance against blackleg but can be prone to breakdown due to race specificity while QR is activated by many minor genes expressed at later stages of plant growth, producing small additive effects of partial resistance which exert less pressure on blackleg pathotypes and therefore considered more durable (Delourme et al. 2014; Marcroft et al. 2012). As BLresp encapsulates both MGR and QR for selection of blackleg resistance, it is therefore important for breeders to consider the presence and efficacy of resistance sources to appropriately perform genotype selection that prolongs the effectiveness of blackleg resistance in commercial varieties.

It is important to note that when the genetic correlation between traits is unity such as WN22 and HS23 (Table S1, Online Resource 2), the estimated genetic variance of BLresp is therefore zero as genotypes ranked the same for both BLeme and BLmat, suggesting no differential responses among genotypes to blackleg disease (Dolferus et al. 2019; Telfer et al. 2021). As the additive component for BLeme was not significant for a majority of datasets, no correlation structure could be fitted for these datasets and hence BLresp could not be derived. Although breeding line selection can be made based on BLmat for these datasets, this presents a shortcoming to the BLresp measure, which can only be applied to scenarios where both traits were significant for additive genetics. This limitation in the novel selection measure BLresp is therefore a current topic of research.

Note that the bivariate model developed in the present study only considers the analysis of individual sites independently. It is therefore desirable to extend this model to accommodate multi-environment trials (MET) which better reflects data from commercial canola breeding programmes and Australia’s NVT system (GRDC 2025). This multi-trait, multi-environment problem requires the consideration of genotype by trait by environment interactions in the modelling of genetic effects (Montesinos-López et al. 2016; Smith et al. 2007) and appropriate selection tools to summarise the predicted genetic effects from this more complex model (Bančič et al. 2023; Smith et al. 2019) for the evaluation of genotype blackleg resistance and its stability across environments. This extension is a current topic of research and would further encourage the adoption of our improved bivariate approach in breeding programmes and NVT.

## Conclusion

This study presents an extended bivariate approach incorporating a genomic relationship matrix which significantly improved model fit across most datasets, reflecting the importance of considering genetic relatedness between genotypes in modern plant breeding programmes. Furthermore, the 2DIMVAR1 residual model significantly improved the modelling of residual error effects in many datasets over the traditional separable models, demonstrating the flexibility in allowing for differing spatial correlations across sampling times. The novel selection measure responsiveness provided a useful and informative summary from the bivariate analysis for the selection of blackleg resistance in canola breeding programmes. The methods from the present study can be easily transferable to other abiotic stress tolerance or resistance studies for genotype selection within breeding programmes. Ultimately, the combination of these innovations for the bivariate analysis of canola blackleg survivability can accelerate genetic gain for blackleg resistance in variety selection trials. As it is desirable to perform variety selection and acquire disease resistance ratings from a series of field trials across multiple years and locations, it is therefore an ongoing research to extend the improved bivariate analysis for MET data.

## Supplementary Information

Below is the link to the electronic supplementary material.

Supplementary file 1 (pdf 24065 KB)

Supplementary file 2 (pdf 109 KB)

Supplementary file 3 (pdf 109 KB)

Supplementary file 4 (r 6 KB)

## Data Availability

The genomic marker and phenotype data analysed in this study are available at Figshare (https://doi.org/10.6084/m9.figshare.29095535.v1).

## Code Availability

The R script to fit the linear mixed models in this paper is provided in the electronic supplementary material.
