# Supplementary material for: Improved bivariate analysis of canola survivability against blackleg disease
Source: Theor Appl Genet. 2025 Aug 22;138(9):225. doi: 10.1007/s00122-025-04993-x (PMC12373551; doi:10.1007/s00122-025-04993-x)
Supplement: Supplementary file 1 — (pdf 24065 KB) [file 122_2025_4993_MOESM1_ESM.pdf]

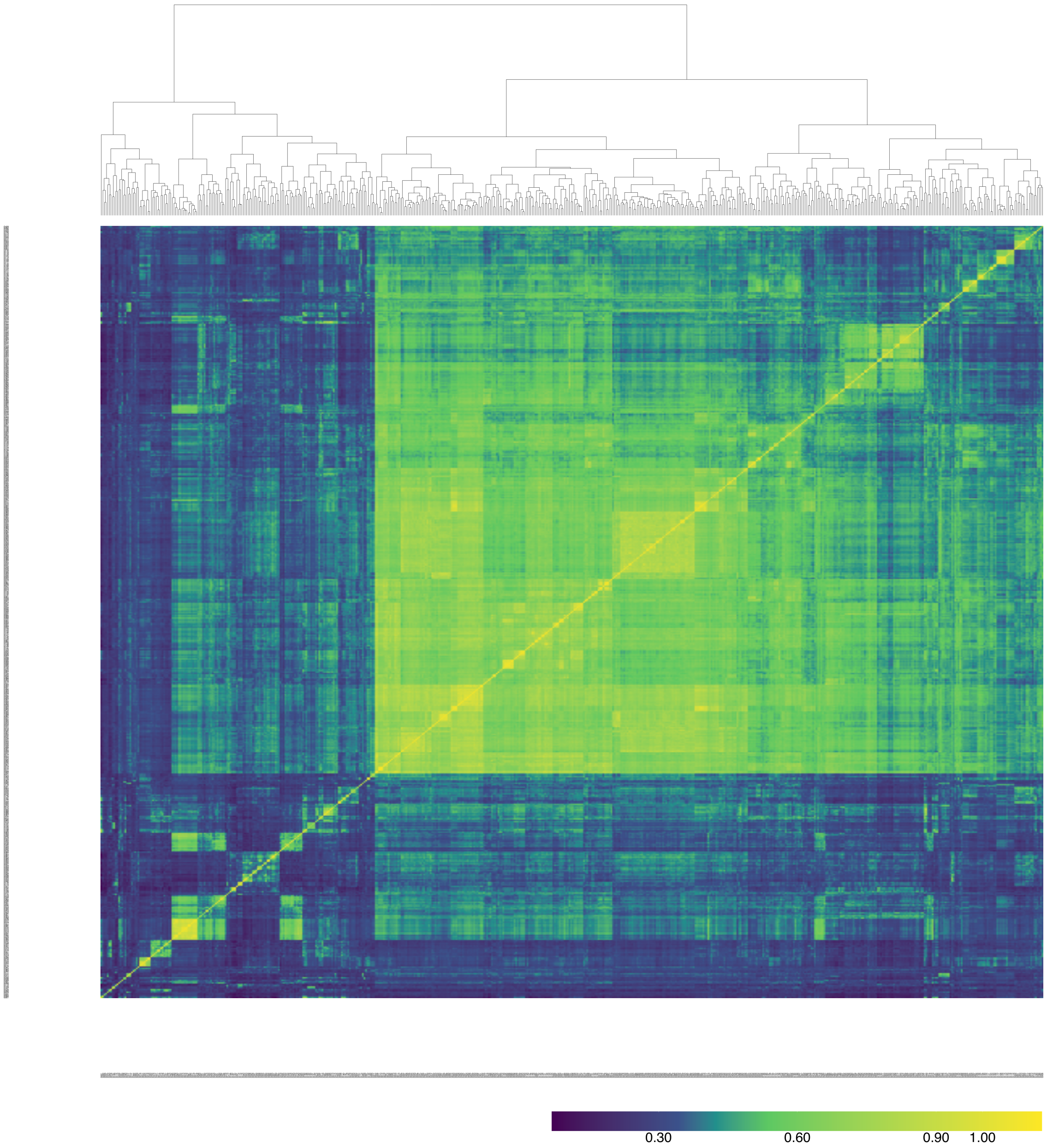

Fig.S1. Heatmap of genomic relationships between all 584 canola genotypes in datasets from 2021 to 2023. Lighter colour asserts that genotypes are more closely related and vice versa for darker colour.
