# Supplementary material for: Improved bivariate analysis of canola survivability against blackleg disease
Source: Theor Appl Genet. 2025 Aug 22;138(9):225. doi: 10.1007/s00122-025-04993-x (PMC12373551; doi:10.1007/s00122-025-04993-x)
Supplement: Supplementary file 2 — (pdf 109 KB) [file 122_2025_4993_MOESM2_ESM.pdf]

Table S1 Summary of selection measures BLresp and BLsur. ρ is the genetic correlation between traits, β is the slope of regression for BLmat against BLeme, σ^2 is the genetic variance of BLresp and Acc is the prediction accuracy of EBLUPs.

	Bivariate BLresp								Univariate BLsur	
	Additive				Non-additive					
Dataset	ρ_a	β_a	σ_a^2	Acc_a	ρ_r	β_r	σ_r^2	Acc_r	Acc_a	Acc_r
HS21	[a]	[a]	[a]	[a]	0.69	0.77	75.96	0.70	0.53	0.69
YK21	[a]	[a]	[a]	[a]	0.95	0.75	16.40	0.50	0.56	0.40
WN22	[a]	[a]	[a]	[a]	1.00	[b]	0.00	[b]	0.75	[c]
HS22	[a]	[a]	[a]	[a]	0.79	0.57	103.77	0.65	0.74	0.58
RS22	[a]	[a]	[a]	[a]	0.81	0.73	0.27	0.66	0.57	0.53
YK22	[a]	[a]	[a]	[a]	0.91	0.77	32.81	0.88	0.17	0.81
WN23	0.79	1.53	0.45	0.38	[a]	[a]	[a]	[a]	0.50	[c]
HS23	1.00	[b]	0.00	[b]	0.35	0.37	0.25	0.58	0.59	0.46
RS23	0.77	0.88	65.45	0.49	0.79	0.67	17.73	0.48	0.49	0.56
YK23	0.63	1.03	187.74	0.55	[a]	[a]	[a]	[a]	0.54	[c]

[a]BLeme and/or BLmat variance component = 0, hence no correlation structure fitted

[b]genetic correlation is unity, hence no deviations around regression line

[c]genetic variance component was on the boundary
