# Supplementary material for: Improved bivariate analysis of canola survivability against blackleg disease
Source: Theor Appl Genet. 2025 Aug 22;138(9):225. doi: 10.1007/s00122-025-04993-x (PMC12373551; doi:10.1007/s00122-025-04993-x)
Supplement: Supplementary file 3 — (pdf 109 KB) [file 122_2025_4993_MOESM3_ESM.pdf]

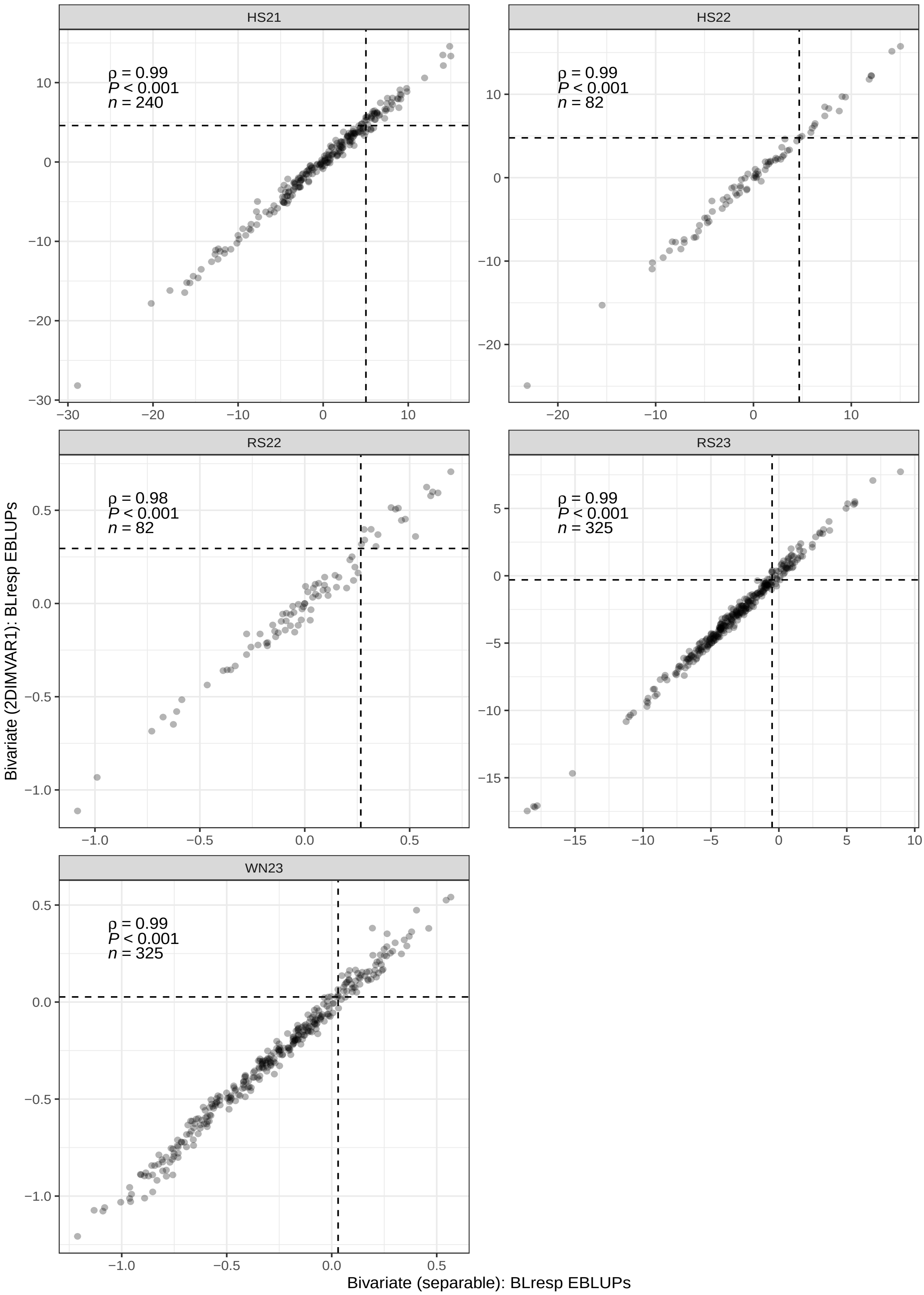

Fig.S2. The EBLUPs of BLresp derived from bivariate genomic model with flexible 2DIMVAR1 residual structure against the historic three-way separable structure. Here, only datasets that showed significant improvement in 2DIMVAR1 model (see Table 4) were included. The cut-off for top 20% of genotypes was indicated with vertical dotted lines for separable and horizontal dotted lines for 2DIMVAR1. ρ represents the Spearman's rank correlation coefficient between the two sets of EBLUPs while n represents the number of genotypes in each dataset. Note: the non-additive EBLUPs were used for datasets HS21, HS22 and RS22 (additive not significant), while additive EBLUPs were used for remaining datasets.
